# Bioremediation of Synthetic Wastewater with Contaminants of Emerging Concern by *Nannochloropsis* sp. and Lipid Production: A Circular Approach

**DOI:** 10.3390/bioengineering12030246

**Published:** 2025-02-28

**Authors:** Bruna Santos, Juliana Araújo, Beatriz Carvalho, Carolina Cotrim, Raul Bernardino, Filomena Freitas, Abílio J. F. N. Sobral, Telma Encarnação

**Affiliations:** 1 PTScience, Rua da Liberdade nº10, 2460-060 Alcobaça, Portugal; bruna.santos@ptscience.pt (B.S.);; 2 Associate Laboratory i4HB—Institute for Health and Bioeconomy, School of Science and Technology, NOVA University Lisbon, 2829-516 Caparica, Portugal; a4406@fct.unl.pt; 3 UCIBIO—Applied Molecular Biosciences Unit, Department of Chemistry, School of Science and Technology, NOVA University Lisbon, 2829-516 Caparica, Portugal; 4 Coimbra Chemistry Centre-Institute of Molecular Sciences (CQC-IMS), Department of Chemistry, University of Coimbra, 3004-535 Coimbra, Portugal; juliana.g.araujo2304@gmail.com (J.A.);; 5 MARE—Marine and Environmental Sciences Centre/ARNET—Aquatic Research Network, ESTM—School of Tourism and Marine Technology, Polytechnic of Leiria, 2520-614 Peniche, Portugal; carolina.cotrim@outlook.com (C.C.);; 6 LSRE-LCM—Laboratory of Separation and Reaction Engineering-Laboratory of Catalysis and Materials, School of Technology and Management (ESTG), Polytechnic Institute of Leiria, 2520-614 Peniche, Portugal; 7 ALiCE—Associate Laboratory in Chemical Engineering, Faculty of Engineering, University of Porto, Rua Dr. Roberto Frias, 4200-465 Porto, Portugal

**Keywords:** microalgae, *Nannochloropsis* sp., contaminants of emerging concern, endocrine disrupting chemicals, bioremediation, lipids, fatty acids

## Abstract

Contaminants of emerging concern (CECs) pose a potential risk to human and environmental health. Microalgae bioremediation is a promising approach for transforming or removing contaminants from the environment, while contributing to the circular economy. In this study, *Nannochloropsis* sp. was effectively used for the simultaneous removal of six CECs: paracetamol, ibuprofen, imidacloprid, methylparaben and bisphenol A at 10 µg mL^−1^ and triclosan at 0.5 µg mL^−1^ from synthetic wastewater, which were able to survive under such concentrations, higher than those commonly found in the environment (up to 2.82 µg mL^−1^ of methylparaben). High removal efficiencies were reached for methylparaben (100%) and bisphenol A (93 ± 2%), while for imidacloprid, paracetamol and ibuprofen, 30 ± 1%, 64 ± 2% and 49 ± 5% were removed, respectively. Subsequently, lipids were extracted, and the FAME profile was characterised using GS-MS. The main fatty acids identified after bioremediation were hexadecadienoic acid isomers (C16:2), palmitic acid (C16), linoleic acid (C18:2) and γ-linolenic acid (C18:3). The absence of oleic acid and stearic acid was noticed, suggesting an alteration in the lipidic profile due to contaminant exposure. By exploring the quantification of fatty acids in future work, potential applications for the extracted lipids can be explored, further demonstrating the feasibility of this circular process.

## 1. Introduction

The discharge of waste materials into the ecosystems, resulting from industrial, agricultural and urban activities, has led to significant environmental pollution, compromising the quality of water, soil and air [1,2,3]. A wide variety of substances of concern are being released and accumulating in the environment, raising concerns about their effects on wildlife and human health [4,5]. These compounds, classified as contaminants of emerging concern (CECs), despite having uncertain effects, may exhibit carcinogenic, mutagenic or endocrine-disrupting activities [3]. Therefore, more research is needed to better understand the sources and fate of these substances. There is also the need to develop additional sustainable technologies to remove these substances and potentially restore the environment [6,7]. Possible technologies include bioremediation, phytoremediation and mycoremediation of wastewater [8,9,10]. These are valuable approaches as they comprise the transformation or removal of contaminants from the environment using living organisms, such as bacteria, fungi, plants and microalgae. Unlike conventional remediation methods, which often involve toxic chemicals and the production of secondary waste, these technologies can offer sustainable and ecologically benign alternatives or complementary approaches [11,12,13].

Microalgae bioremediation has significant advantages, including high growth rates and high versatility [14,15,16]. Several microalgae species have been studied for wastewater purposes [17,18,19,20,21,22,23]. These organisms were shown to be highly effective in the removal of nitrogen and phosphorus from wastewater, with these nutrients then being used for growth [24]. The species *Chlamydomonas reinhardii* [18], *Chlorella vulgaris* [19], *Dunaliella salina* [20], *Isochrysis galbana* [21], *Nannochloropsis* sp. [22] and *Spirulina* sp. [23] were reported to be suitable for the removal of pollutants from water, which included heavy metals, pharmaceuticals and pesticides [14].

Another significant advantage of using microalgae for bioremediation is the possibility of further utilising the generated biomass to extract valuable bioproducts, in accordance with the circular economy concept [25]. Circular economy promotes the reuse of materials that are already present in nature, thereby ensuring that no resources are wasted, and all elements can be utilised for alternative purposes. This approach serves to minimise the wastage of resources and control pollution [26].

Consequently, there is a growing interest in investigating microalgae for bioremediation purposes, in order to regenerate nature while simultaneously generating value. In the present microalgae bioremediation study, *Nannochloropsis* sp. was selected due to its characteristics, including rapid growth, adaptability, resilience and the ability to accumulate substantial amounts of lipids [22]. 

*Nannochloropsis* is a genus of small unicellular marine microalgae, known for their ability to accumulate more than 60% of their dry weight in the form of lipids, mainly triacylglycerides [27,28,29,30,31]. For this reason, they are considered an oleaginous species, recognised for their high lipid production, and are a promising resource for a wide range of applications such as surfactants, resins, binders, biofuel and biopolymers [32,33,34,35,36,37]. Some *Nannochloropsis* species were reported for the bioremediation of different contaminants, though limited literature is available in this area, particularly regarding the simultaneous removal of multiple CECs [38,39,40]. Therefore, for this study, six pollutants were selected as representatives of important CEC categories, namely pharmaceuticals, pesticides, plasticisers and household and personal care products [2]. They all represent a concern to the environment due to their toxicity, occurrence and persistence in the environment and/or endocrine-disrupting activity. The selected pollutants were paracetamol (PAR), bisphenol A (BPA), imidacloprid (IMID), methylparaben (MP), triclosan (TCS) and ibuprofen (IBU). Table 1 shows the relevant characteristics and physicochemical properties of each pollutant.

Acetaminophen, commonly known as PAR, is a non-steroidal anti-inflammatory drug, whose long-term exposure, even at low concentrations, can have negative effects on aquatic ecosystems, including DNA and reproductive system damage, oxidative stress and other associated pathologies [41,42].

IBU, another non-steroidal anti-inflammatory drug, may have long-term negative consequences for aquatic animals and other marine creatures, including cytotoxic and genotoxic effects, high oxidative stress and adverse impacts on growth rate, reproduction and behaviour. The characteristics of IBU, including its insolubility in water, high lipophilicity and resistance to degradation are of major concern [41,42,43].

BPA is a synthetic chemical, mostly used to manufacture polycarbonate plastics and resins. It is a compound that has gained increased awareness due to its identification by the European Union as an endocrine-disrupting chemical. Some effects of exposure to this compound have been reported in both humans and animals, with symptoms including polycystic ovary syndrome, neurological disorders, cancer, thyroid dysfunction and diabetes. Adverse reproductive system development and infertility have also been observed, which can be considered a consequence of its endocrine-disrupting activity [44,45,46].

MP is a synthetic preservative widely used in cosmetics [47]. Based on current data, the structure of MP mimics oestrogen molecules and causes endocrine disruption. In addition, it may cause other acute toxic effects, including negative effects on growth, oxidative stress, cancer development and nervous system malfunctions [48,49,50].

TCS is an antiseptic, antibacterial and antifungal chemical used as preservative agent in the cosmetic industry. Studies showed that this compound is acutely toxic to living beings, affecting numerous aquatic organisms, with changes in physiology and biochemistry, and negative effects on development and reproduction. Its structural similarity to the thyroid hormone can disrupt the endocrine system, including androgenic and estrogenic activity [51,52,53].

IMID is a neonicotinoid insecticide primarily employed in agriculture to combat pests. Its excessive use causes releases into the environment, which can be severely harmful to beneficial species, including honeybees and other pollinators. IMID is highly toxic, stable and soluble in water. It acts on the central nervous system of insects, causing blockage of the nicotinergic neuronal pathway, leading to death. It is also considered potentially dangerous to aquatic species, humans and terrestrial animals [22,54,55,56].

Regarding the selected CECs, limited literature is available on their bioremediation using microalgae. Microalgae species of the genus *Chlorella*, *Chlamydomonas* and *Scenedesmus* are the most commonly reported for bioremediation of CECs [17]. For instance, Ji et al. (2014) reported the removal of 24% of BPA by *Chlorella vulgaris* [57]. Microalgae-based remediation mechanisms include bioadsorption, bioaccumulation and biodegradation [25]. A major challenge in using microalgae bioremediation as a strategy for the removal of CECs is the vast diversity of these molecules and the difficulty in screening suitable and efficient microalgae species.

The aim of this study is to investigate the bioremediation potential of *Nannochloropsis* sp., in the context of the circular economy; more specifically, it is to assess the removal quantifying six selected CECs and characterise the microalgae lipids produced. The effect of those CECs on the accumulation of lipids produced and the fatty acids profile were assessed.

## 2. Materials and Methods

### 2.1. Reagents and Chemicals

PAR and TCS were purchased from Fagron Iberica (Barcelona, Spain) and ibuprofen was supplied by Laboratórios Medinfar (Lisboa, Portugal). Imidacloprid was acquired by Ehrenstorfer™ (Augsburg, Germany) and bisphenol A from Sigma-Aldrich (Steinheim, Germany). Methylparaben was purchased from the Tokyo Chemical Industry (Zwijndrecht, Belgium). The microalgae *Nannochloropsis* sp. and the commercial formulation Cell-hi TEViT was obtained from Varicon Aqua Solution (Malvern, UK). This is based on the f2 culture medium without nitrates and phosphates [56]. All other reagents and solvents were of analytical or HPLC grade.

### 2.2. Culture Conditions

The initial culture was grown under photoautotrophic conditions in a 10 L round flask in an f2 growth medium, composed of 25 g L^−1^ NaCl, 0.3 g L^−1^ NaNO_3_, 0.00565 g L^−1^ KH_2_PO_4_ and 0.063 g L^−1^ TE-ViT [41,58,59]. The culture was aerated by bubbling atmospheric air at a rate of 1500 cm^3^ min^−1^, and sunlight was used as the light source. The culture was kept at room temperature, being used as inoculum, when it reached the desired cellular density (≈1.30 × 10^6^ cell mL^−1^).

### 2.3. Experimental Design

Preliminary studies were performed using concentrations of 10, 25 and 50 µg mL^−1^ of the different pollutants in a mixture, except TCS, which due to its low solubility, was tested at 0.5, 20 and 22.5 µg mL^−1^, respectively. In parallel, individual experiments were also performed for each pollutant at 50 µg mL^−1^, to understand if any particular CEC significantly influenced the growth of *Nannochloropsis* sp. The preliminary studies were conducted in 250 mL Erlenmeyer flasks, with a working volume of 200 mL, sealed with Parafilm^®^. From these studies, it was noticed that the medium was leaking from the Parafilm^®^ during sampling, likely due to condensation or high aeration. To prevent the high losses of water noticed during the experiments due to inadequate isolation of the Erlenmeyer flasks with Parafilm^®^, the subsequent experiments were performed in water bottles with screw caps. This proved to be more effective in controlling evaporation.

**Figure 1:**
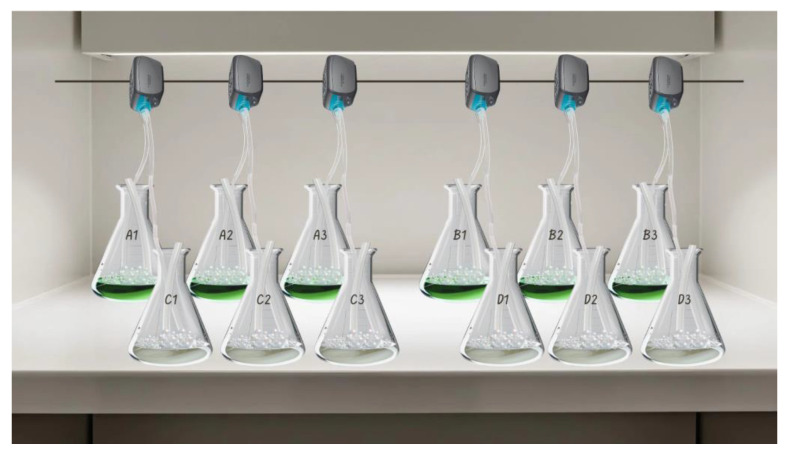
Schematic representation of the experimental setup. A1, A2, A3—*Nannochloropsis* sp. In the f2 medium; B1, B2, B3—*Nannochloropsis* sp. in the f2 medium with pollutants; C1, C2, C3—in the f2 medium with CECs; D1, D2, D3—CECs.

In the following experiments, three factors were evaluated for their influence in the removal of the six selected contaminants: abiotic factors, culture medium and microalgae bioremediation. For such purposes, four experimental groups were prepared in triplicate, with a total of 12 experimental units. The first group was designated as negative control (A: microalgae in f2 medium without CECs), the second group was the bioremediation experiment (B: microalgae in f2 with CECs), the third group was a positive control to evaluate the medium influence in the removal of the contaminants (C: CECs in f2 medium) and the fourth group was a second positive control to analyse the effect of abiotic factors (D: CECs in distilled water). The experimental setup is illustrated in Figure 1.

The bioremediation experiments were performed for 10 days using 500 mL water bottles as bioreactors. A working volume of 200 mL was found appropriate for the study. For PAR, MP, IMID, BPA and IBU, concentrations were set at 10 µg mL^−1^, and for TCS, a concentration of 0.5 µg mL^−1^ was used. The CEC concentrations were selected to evaluate the response of the microalgae cells under a higher stress than those found in the environment (the highest reported was 2.82 µg mL^−1^ for MP [58]). In addition, the values were chosen to ensure analytical accuracy and reliability.

Approximately 1.3 × 10^6^ cell mL^−1^ of *Nannochloropsis* sp. were inoculated in each experiment. The experiments were carried out with a 24:0 light/dark photoperiod, with an irradiance of 45-55 µmol m^−2^ s^−1^. The temperature was maintained at 26 ± 2 °C throughout the experiment. Distilled water was added when necessary to ensure the same constant volume, compensating for water loss through evaporation. Samples of 5 mL were collected at 24 h intervals and stored at −20 °C until analysis. Each experiment was carried out in triplicate.

### 2.4. Instrumentation and RP-HPLC Conditions

The quantification of PAR, IBU, BPA, MP, TCS and IMID was carried out using a Dionex Ultimate 3000 system (Germering, Germany) equipped with an auto-injector and four variable U*V*/*v*isible dual-wavelength detectors (DAD). The column used for the analysis was a Kinetex^®^ Biphenyl, Phenomenex^®^ (Torrance, CA, USA), with 5 μm particle size, 100 Å pore size, 4.6 mm internal diameter and 250 mm length, supported with a SecurityGuard™ cartridge Phenomenex^®^ (Torrance, CA, USA), with 4.6 mm internal diameter, which was in an oven at a temperature of 40 °C. The results were acquired and processed using Chromeleon 7 software (Version 7.2.10 (23925)). A RP-HPLC-validated method, developed for this specific purpose, was used [56]. The mobile phase consisted of a mixture of the organic solvent methanol with 5 mM of ammonium acetate with 0.1% acetic acid (Eluent A), and a buffer, ultra-pure water with 5 mM of ammonium acetate with 0.1% acetic acid (Eluent B), at a constant flow rate of 0.6 mL min^−1^. Chromatographic analysis was conducted in multistep gradient mode according to Table 2.

### 2.5. Removal Strategies

The concentration of each contaminant was assessed using the developed RP-HPLC method and calculated using the respective calibration curves. The decrease in the concentration of each contaminant was calculated to assess the removal efficiencies of the different removal strategies.

Total removal (%) was calculated using Equation (1), where *Ci* is the initial concentration of the contaminant and *CBf* is the final concentration in experiment B:

%Total Removal = (*Ci* − *CBf*)/*Ci* × 100 (1)

The abiotic removal (%) was calculated using Equation (2), where *CDf* is the final concentration in experiment D:

%Abiotic Removal = (*Ci* − *CDf*)/*Ci* × 100 (2)

The intervention the culture medium in the removal of the CECs (%) was calculated using Equation (3), where *CCf* is the final concentration in the experiment C:

%Medium Interaction = ((*Ci* − *CCf*) − (*Ci* − *CDf*))/*Ci* × 100 (3)

The efficiency of microalgae bioremediation (%) was calculated using Equation (4):

%Microalgae Bioremediation = ((*Ci* − *CBf*) − (*Ci* − *CCf)*)/*Ci* × 100 (4)

### 2.6. Cell Counting

A haemocytometer (Neubauer chamber) was used under an optical microscope to determine cell density during the bioremediation experiments. 

### 2.7. Lipid Extraction

For the extraction of lipids from *Nannochloropsis* sp. the biomass obtained after the 10 days of the bioremediation experiments was extracted following the Bligh and Dyer method [60,61]. Briefly, the biomass obtained in experiments A and B was washed, concentrated by centrifugation and lyophilized. Chloroform, methanol and water in a ratio of 2:2:1.8 was added to the dried biomass and the mixture was vortexed, sonicated (10 min) and centrifuged for 5 min at 1200× *g*. The chloroform phase was transferred to a new tube and 1 mL chloroform was added. It was again vortexed, sonicated and centrifuged under the same conditions. The chloroform phase was extracted again and, finally, the chloroform was evaporated from the sample for the gravimetric quantification of the extracted lipids, which were stored at −20 °C.

### 2.8. Transesterification of Lipids into FAME

To analyse the profile of fatty acid methyl esters (FAME) [60], the extracted lipids were treated with 3 mL of methanol containing 5% (*v*/*v*) sulfuric acid for 3 h at 70 °C. Thereafter, the samples were cooled to room temperature and 3 mL of MilliQ water and 3 mL of n-hexane (Sigma Aldrich, 99.9%) were added. The samples were then subjected to an agitation process using a vortex for 5, followed by a mixing procedure for 15 min, employing a tube rotator. The samples were centrifuged for 5 min at 1200× *g* and the hexane phase (2 mL) was collected into a fresh glass tube. MilliQ water (2 mL) was added to wash the sample, and a further cycle of vortex agitation for 5 s and centrifugation at 1200× *g* for 5 min was conducted. Finally, the obtained FAMEs were stored at −19 °C, under a nitrogen gas atmosphere, until analysis.

### 2.9. GC-MS Analysis of FAMEs

The GC-MS system used was an Agilent Technologies 7820A GC System equipped with MS Agilent Technologies 5975 Series MDS. An HP-5ms Agilent fused silica capillary column, 30 m × 0.25 mm × 0.25 µm, was used. The column temperature programme was set as follows: 60 °C hold for 3 min, raise at 10 °C min^−1^ to 250 °C, hold for 10 min, raise at 5 °C min^−1^ to 280 °C and hold for 5 min. The total run time was 43 min.

The GC injector was held isothermally at 250 °C with a spitless mode. The solvent delay time was set at 3 min. Helium was used as the carrier gas at a flow rate of 1 mL min^−1^ by using flow control. The GC-MS interface temperature was maintained at 280 °C. The quadrupole temperature was 150 °C.

The MS instrument was operated in electron impact (EI) ionisation mode with an electron energy of 70 eV, and the scan ranged from 50 to 500 amu in scan mode.

### 2.10. Statistical Analysis

Statistical analysis was performed by one-way ANOVA and Student’s *T*-test. Significant differences were considered for a *p* < 0.05.

## 3. Results and Discussion

### 3.1. Preliminary Results

**Figure 2:**
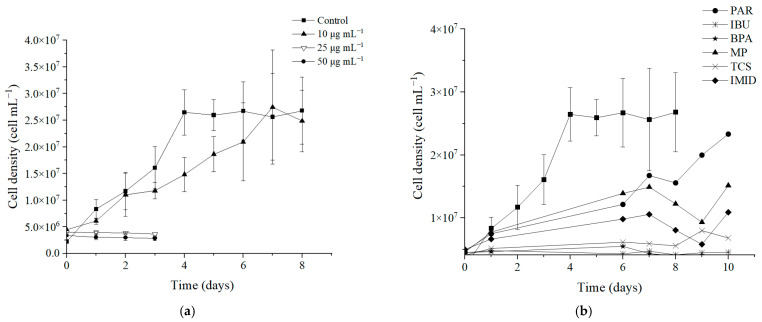
(**a**) Cell density obtained from bioremediation experiments in the negative control, and *Nannochloropsis* sp. spiked with 10 µg mL^−1^, 25 µg mL^−1^ and 50 µg mL^−1^ of the mixture solution (TCS at 10, 20 and 22.5 µg mL^−1^). (**b**) Cell density obtained from bioremediation experiments of each individual contaminant at 50 µg mL^−1^ (TCS at 22.5 µg mL^−1^). Vertical bars represent standard error. (Paracetamol (PAR), bisphenol A (BPA), imidacloprid (IMID), methylparaben (MP), triclosan (TCS) and ibuprofen (IBU)).

Preliminary experiments (Figure 2a) were performed before bioremediation studies, in order to explore the limits of resistance exhibited by *Nannochloropsis* sp. cells to extreme concentrations (10, 25, 50 µg mL^−1^) of CECs in the mixture (except TCS at 0.5, 20 and 22.5 µg mL^−1^ respectively), which may occur in wastewater discharge sites or as a consequence of accidental spills, resulting in environmental accumulation. The individual experiments at 50 µg mL^−1^ (TCS at 22.5 µg mL^−1^) aimed to identify any specific CEC more toxic for *Nannochloropsis* sp. cells. Note that, for simplicity, the concentrations are generally given as 10, 25 or 50 µg mL^−1^ throughout the text, but the TCS concentrations are lower, as mentioned above, and should be considered as 0.5, 20 or 22.5 µg mL^−1^, respectively.

As shown by the profiles presented in Figure 2b, the presence of the contaminants in the medium at a concentration of 10 µg mL^−1^ had a significant impact on *Nannochloropsis* sp. cellular growth. Nevertheless, within 8 days in the presence of the CECs’ mixture, the culture was able to mitigate the pollutants’ toxicity, achieving a cell density comparable to that of the control group. It is reported that the antioxidant enzymes effectively regulate the ROS produced in the presence of pollutants, protecting cells from oxidative damage [62]. Therefore, it can be concluded that, at the tested concentration, the pollutants did not inhibit the overall cellular growth, despite causing slow cell growth. However, this behaviour was not verified at higher concentrations of the mixture. *Nannochloropsis* sp. cells spiked with the mixture at 25 and 50 µg mL^−1^ were unable to survive such concentrations, which resulted in significant oxidative damage, leading to complete growth inhibition during the 3-day experiment. Similar data have been reported in the literature and are explained by the excessive production of ROS, triggered by the exposure to CECs above levels tolerable for the cells [63,64]. Therefore, a dose–response relationship can be deduced from these preliminary experiments, as the higher the CECs concentration, the greater their negative effect on *Nannochloropsis* sp. cell growth.

Upon evaluation of each pollutant individually (Figure 2b), at 50 µg mL^−1^ for 10 days, different effects in *Nannochloropsis* sp. cell growth by each CEC could be observed. Thus, it can be suggested that, at the working concentration, the CECs’ toxicity to these microalgae has the following order: PAR < MP < IMID < TCS < IBU < BPA.

**Figure 3:**
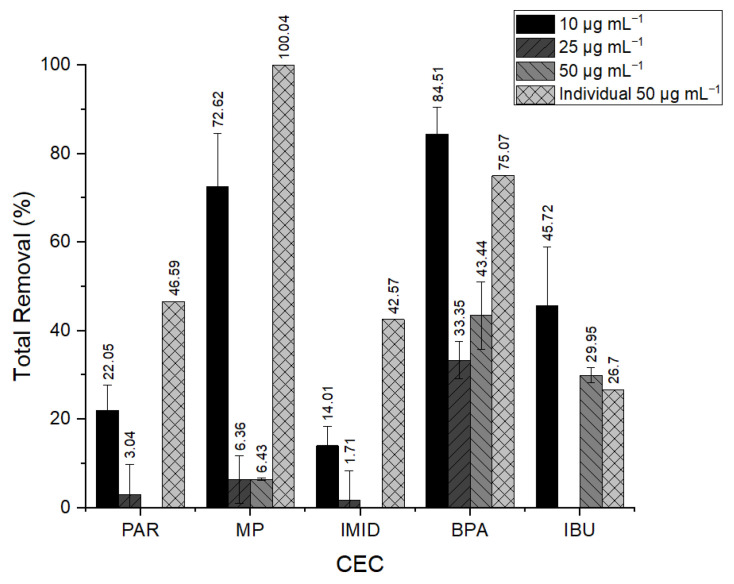
Removal efficiency obtained for each contaminant in each experimental condition (10 µg mL^−1^, 25 µg mL^−1^, 50 µg mL^−1^, individual at 50 µg mL^−1^). (Paracetamol (PAR), bisphenol A (BPA), imidacloprid (IMID), methylparaben (MP), triclosan (TCS) and ibuprofen (IBU)).

In terms of contaminants’ removal, each experimental condition resulted in different efficiency, as represented in Figure 3. Although the variation in experiment duration due to cell death, the removal efficiencies observed were also dependent on the initial concentration and the presence of other contaminants.

As shown in Figure 3, it is noticeable that individual experiments achieved higher removal efficiency values, with the exception of IBU. Thus, by comparing the efficiency of the removal of individual CECs with the mixture at the same concentration, it can be concluded that the combined presence of the contaminants reduces the efficiency of the overall removal. This outcome may be related to the different experimental durations. The experiment lengths varied due to *Nannochloropsis* sp. cell viability, which decreased with increasing CEC concentrations. This may have influenced removal efficiencies due to exposure to removal strategies for different lengths of time.

After individual experiments, the mixture containing 10 µg mL^−1^ of each contaminant achieved a higher removal and was chosen for the subsequent experiments.

### 3.2. Bioremediation Experiments

#### 3.2.1. Population Density

**Figure 4:**
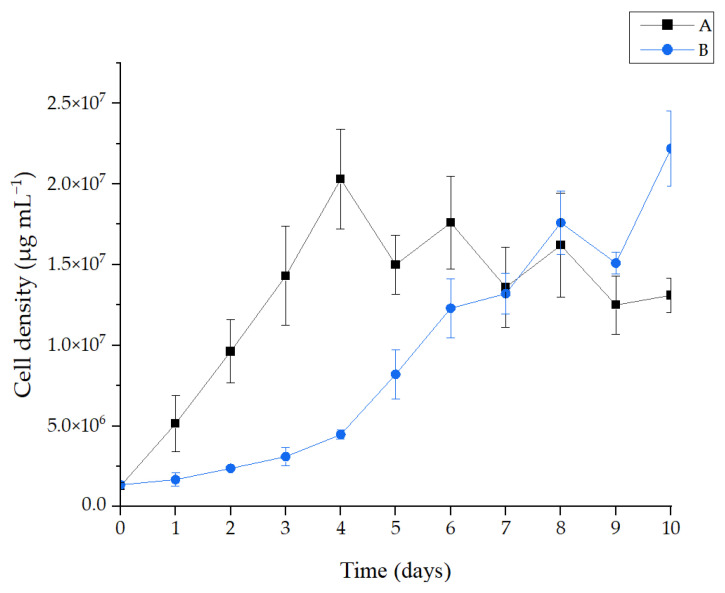
Cell growth of *Nannochloropsis* sp. in culture A (without pollutants) and culture B (with PAR, IBU, BPA, MP and IMID at 10 μg mL^−1^ each, and TCS at 0.5 μg mL^−1^) for 10 days. (Paracetamol (PAR), bisphenol A (BPA), imidacloprid (IMID), methylparaben (MP), triclosan (TCS) and ibuprofen (IBU)).

Culture A grew from 1.28 × 10^6^ cells mL^−1^ to 2.03 × 10^7^ cells mL^−1^ in the first four days and remained stable thereafter, with minor changes until the last day of the test. On the other hand, culture B grew from 1.35 × 10^6^ cells mL^−1^ to 2.22 × 10^7^ cells mL^−1^ after 10 days. This culture, unlike the culture without contaminants, remained in constant growth until the last day of the test. The highest cell density was 2.22 × 10^7^ cells mL^−1^ obtained in microalgae culture B on the last day of the experimental trial. At the end of the same period, the microalgae in the absence of contaminants had a cell density of 1.31 × 10^7^ cells mL^−1^. This variation in density between cultures can also be seen in the differences in the intensity of the green colour, where a darker green indicates a higher concentration of microalgae (experiment B) and a lighter green a lower concentration of microalgae (experiment A). Figure 4 shows similar cell densities were reached in both experiments on the seventh day of cultivation, when culture A had a cell density of 1.36 × 10^7^ cells mL^−1^ and culture B had a cell density of 1.32 × 10^7^ cells mL^−1^.

It is important to note that the growth of microalgae depends on various factors, such as light intensity, temperature, pH and CO_2_ and O_2_ concentrations. The balance between those factors allows for the healthy development of these microorganisms [65]. That said, during these experimental activities, all the systems were exposed to the same intensity of light, temperature and air flow.

Also, studies have shown that growth is directly related to the biosynthesis of chlorophyll [64]. The presence of contaminants destabilises the photosynthetic processes, reducing energy production and, consequently, causing an imbalance in cell growth [66]. Other studies associate a decrease in the cell growth rate with lower metabolic activity in the presence of toxic compounds. Slower growth is also related to lower nutrients’ absorptions from the medium [67].

When interpreting these results, it should be noted from the outset that these microalgae cultivation experiments consist of a batch culture. In a batch culture, there is a single inoculation of microalgae, followed by a growth period of several days and finally, the algae population reaches its maximum or near maximum density [68]. It is interesting to note that there is exponential growth, faster in culture A than in culture B, followed by a stabilisation of the population density, only in culture A. This may be due to the limited nutrients available for the growth of these organisms. In culture B, it is assumed that there is a phase of adaptation of the microalgae to the disturbance they cause in the algal environment. This adaptation phase suggests that the algae did not proliferate as quickly as in culture A. However, despite taking longer (due to the presence of CECs), the culture reached higher cell density; this reveals the microalgae’s ability to survive in the presence of contaminants.

There are some studies in the literature on this subject, but they evaluate the pollutants used in this study separately or in different mixtures of pollutants other than the one used in this work. Thus, studies such as the one by Mohy El-Din, report that the growth of *Nannochloropsis* grown in the f2 medium with a BPA concentration of 10 mg L^−1^ is slightly affected during the first 4 days; however, it improved after that to a specific level by the end of the experiment, at 10 days [63]. This profile resembles the profile obtained for culture B, despite having a slower cell growth rate, which ended up exceeding the growth of culture A.

#### 3.2.2. Removal of CECs

In this study, the microalgae *Nannochloropsis* sp. was exposed to 10 µg mL^−1^ of PAR, MP, IMID, BPA and IBU, and to 0.5 µg mL^−1^ of TCS. Although the theoretical initial concentrations were as defined above, the actual concentrations measured were different due to the compounds’ differing solubility in an aqueous medium. This is a limitation of the method used, which can be overcome by pre-dissolving hydrophobic compounds in an appropriate solvent before adding to the aqueous medium. The removal of each contaminant was assessed by monitoring their concentration throughout the experiments, using a validated RP-HPLC method. The quantification of TCS was not possible using the chromatographic technique which can be explained by its low solubility (Table 1) and difficulty in dissolving in aqueous media.

**Figure 5:**
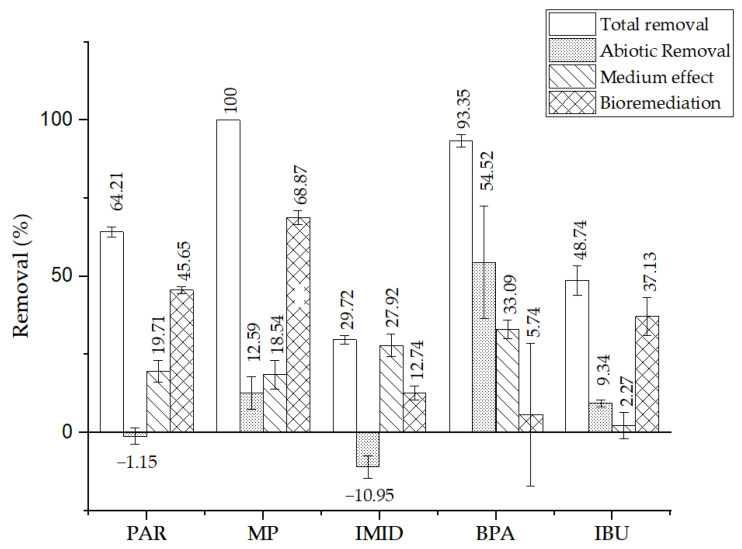
Total removal of PAR, MP, IMID, BPA and IBU, and respective efficiency of removal strategies: abiotic removal, medium effect and bioremediation. (Paracetamol (PAR), bisphenol A (BPA), imidacloprid (IMID), methylparaben (MP), triclosan (TCS) and ibuprofen (IBU)).

The total removal value sums up the effect of bioremediation and external factors such as abiotic effects and the influence of culture medium. The total removal percentage was calculated using Equation (1) and the results are summarised in Figure 5. All measurable CECs were significantly removed to some extent (*p* < 0.05). A full removal of MP (100%) from an initial concentration of 10.43 µg mL^−1^ was achieved by day 5, which was the highest efficiency obtained in this experiment, followed by BPA with a total removal of 93 ± 2% from an initial concentration of 3.70 µg mL^−1^. On the other hand, the lowest removal was calculated for IMID, with a removal efficiency of 30 ± 1% from an initial concentration of 10.30 µg mL^−1^. PAR and IBU reached total removals of 64 ± 2% and 49 ± 5% from initial concentrations of 11.16 µg mL^−1^ and 8.03 µg mL^−1^, respectively.

In the context of microalgae bioremediation, the overall removal efficiency depends on multiple factors, including microalgae species, physico-chemical characteristics of contaminants, initial concentrations, operational conditions and duration of the experiment [25]. When compared with the preliminary experiments using similar initial concentrations, it can be concluded that the change in the setup approach provided improved results. From another perspective, the focus of literature reports is on the removal of individual CECs by different microalgae. For instance, in the present findings, *Chlorella vulgaris* removed 100% of MP, while *P. tricornutum* reached a removal efficiency of 34.2% of the same contaminant, in six days [69]. For BPA, Atengueño-Reyes et al. [70] reported that a consortium of microalgae removed 95.1% from an initial concentration of 16.17 µg mL^−1^, slightly higher than our results.

Reports on the removal of IMID by microalgae are limited. However, a higher removal was obtained for the same microalgae in a study by Encarnação et al. [35], who reported the removal of approximately 50% of this neonicotinoid from an initial concentration of 9.59 µg mL^−1^. This suggests that the presence of multiple contaminants may influence the CEC removal. In the case of PAR, the total removals of this pharmaceutical reached 31.50% from an initial concentration of 50 µg mL^−1^ in the presence of *Cocccomyxa subellipsoidea* during 10 days [71] and 41% from 25 µg mL^−1^ in the presence of *Chlorella sorokininana* during 8 days [72]; both of these are values lower than those observed in this study. Finally, the literature reports a removal percentage of 51.3%, obtained by Jiménez-Bambague et al., for the removal of 1 µg mL^−1^ of IBU in an algal system at bench scale with *Parachlorella kessleri* [64]. Despite the many variables to be taken into account, the ability of the *Nannochloropsis* sp. system to achieve similar or even higher removal percentages in a mixture of pollutants, as other species remove individual CECs, highlights its potential for effective bioremediation in multiple contexts.

It was possible to calculate the efficiency by which the CECs were mostly removed: abiotic removal, medium effect and bioremediation [64]. Figure 5 represents the obtained results. A significant difference in concentrations between the first and last day of experiment D for MP, BPA and IBU (*p* < 0.05) suggests the effect of abiotic factors in the removal of these three CECs. Photodegradation is the abiotic process most likely responsible for the removal of these pollutants. Direct photolysis can degrade compounds susceptible to absorb light at wavelengths above 190 nm, which can explain the removal of 12 ± 5, 54 ± 2 and 9 ± 1% of MP, BPA and IBU in the control D. Abiotic removal was not significant for PAR or IMID (*p* < 0.05). The design of the present bioremediation experiments was based on preliminary studies to minimise identified errors. The negative values in the abiotic removal of PAR and IMID, could be explained by the possibility of the presence of degradation products from the other CECs. These molecules may co-elute and interfere with chromatographic peaks of PAR and IMID giving the appearance of higher final concentrations than the real values. The same effect was previously reported [73]. 

Photodegradation is often enhanced by the presence of reactive oxygen species (ROS), which stimulate the photo-oxidation of compounds in the presence of light. In this study, a statistically significant increase in the degradation of all compounds was observed in the f2 medium (control C) (*p* < 0.05). According to the literature, Fe ions and EDTA, which are constituents of the medium, can stimulate the formation of ROS, thus explaining the obtained results [74]. BPA was the contaminant mostly affected by the medium, with a removal of 33 ± 15%.

**Figure 6:**
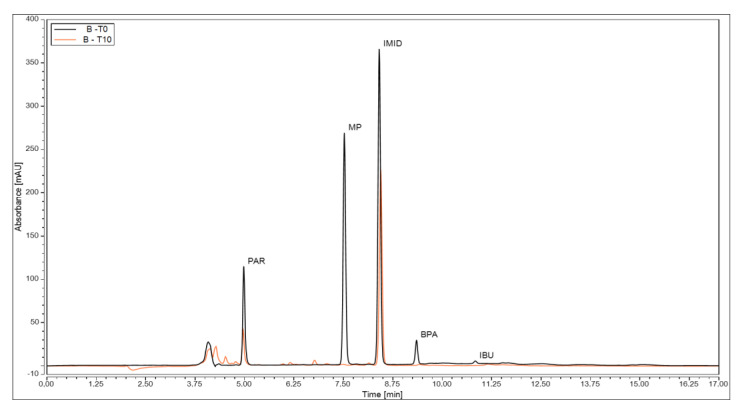
Overlapping of the chromatograms of experiment B on day 0 (black line) and on day 10 (orange line). (Paracetamol (PAR), bisphenol A (BPA), imidacloprid (IMID), methylparaben (MP), triclosan (TCS) and ibuprofen (IBU)).

One of the hypotheses of this study is that experiment B, i.e., the presence of *Nannochlorospsis* sp., would have a significant effect on the removal of the contaminants, thus achieving lower final concentrations than the control experiments C and D. The chromatograms of experiment B (Figure 6) show a reduction in the five peaks after 10 days, and the appearance of small unidentified peaks, suggesting degradation products resulting from biodegradation.

From the total removal of all CECs, a significant percentage was removed by *Nannochloropsis* sp. bioremediation, for a *p* < 0.05, with the exception of BPA, showing a great influence of this microalgae in removal efficiency. Bioremediation played a major role in removing MP, achieving 69 ± 2% of its total removal. PAR and IBU were also significantly removed by *Nannochloropsis* sp., with a removal efficiency of 46 ± 1 and 37 ± 6%, respectively. A lower but still significant reduction was observed for IMID (13 ± 2%).

The available literature referring to the ability of *Nannochloropsis* sp. to remove contaminants from water is limited [40,75,76]. Microalgae can adopt different strategies to perform bioremediation: bioadsortion, bioaccumulation and biodegradation. Biodegradation stands out as it allows for the complete biomineralization of pollutants or their transformation into simpler or less toxic compounds, using enzymatic reactions. In this work, a preliminary assessment of the contaminants that were internalised and bioaccumulated was performed by RP-HPLC. The aqueous supernatant resulting from biomass disruption in lipid extraction and the aqueous phase resulting from lipid transesterification were analysed. PAR, MP and IBU were not identified or quantified in any sample, implying that the *Nannochloropsis* sp. cells were able to break down the compounds into metabolites that could not be measured in this study. The appearance of novel peaks in the chromatogram of experiment B, on day 10 (Figure 6), may also suggest the presence of degradation products in the culture medium. IMID and BPA, on the other hand, were found in the aqueous phase of the lipid extract, at 0.54 ± 0.03 and 3.28 ± 0.51 µg mL^−1^, respectively, suggesting their bioaccumulation by the microalgae. The efficiency of biodegradation largely depends on the complexity of the compound’s structure. Compounds with complex, cyclic structures are less prone to biodegradation, whereas linear, non-saturated and electron-donating groups are easier to transform [76]. This may be a plausible explanation for the preferential degradation of PAR, MP and IBU, with only one aromatic ring, which are less complex than IMID and BPA with two cyclic structures.

In terms of bioaccumulation, the compound BPA was detected at concentrations above those expected, according to Equation (4) (bioremediation was not significant). This equation considers that the weight of the removal mechanisms is always the same in the absence (experiment C) and in the presence of microalgae cells (experiment B). However, according to the obtained data, it can be suggested that, in practice, the bioremediation mechanism suppresses the abiotic pathways. Thus, BPA molecules may be removed and internalised mainly by microalgae cells, and the impact of photodegradation is attenuated in this scenario. In addition, studies for BPA bioremediation from other studies report that the predominant removal pathway is biodegradation and bioaccumulation by microalgae, which is consistent with the findings of the present study [57]. However, this remains hypothetical, and further studies are required to confirm these findings.

### 3.3. Nannochloropsis sp. Biomass Valorisation

#### 3.3.1. Biomass and Lipid Yield

The microalgae genus *Nannochloropsis* is classified as an oleaginous organism due to its ability to accumulate large quantities of lipids [29]. In the context of the circular economy, this characteristic makes it possible to reduce the cost of wastewater treatment by exploring subsequent biomass valorisation, focusing on lipid extraction [39]. In this work, biomass yield, lipid yield and %DW of lipids in samples A and B were evaluated to understand the effect of the CECs on biomass production and lipid synthesis from *Nannochloropsis* sp.

**Figure 7:**
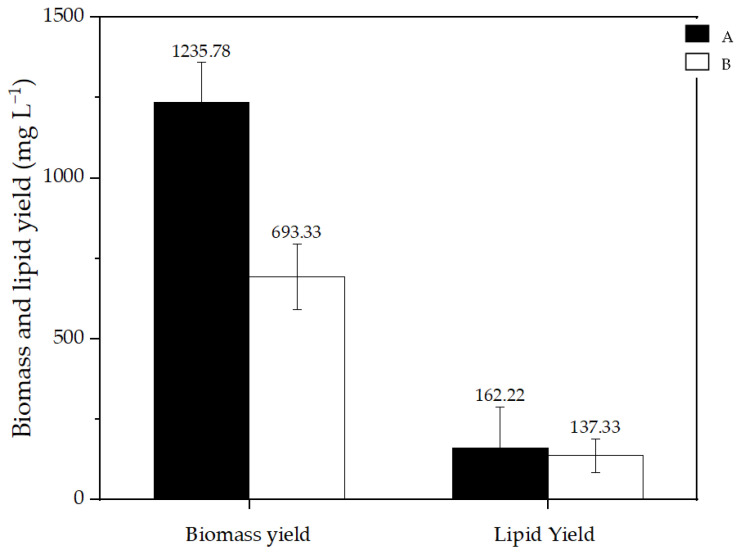
Biomass and lipid yield in *Nannochloropsis* sp. from experiments A and B.

Figure 7 shows significant differences in biomass yield between the samples (*p* < 0.05). The biomass yield was lower in the presence of the contaminants (sample B), demonstrating that despite achieving higher cell densities (see Section 3.2.1), the weight and composition of the cells might be affected by this stress factor [77].

On the other hand, lipid yields were found to be similar in both samples, meaning that in terms of %DW, lipids in experiment B (21 ± 11%) were higher than in experiment A (12 ± 9%). These data were, however, not found statistically significant due to high SD values, which can be explained by the need to adapt and optimise the extraction protocol. Bligh and Dyer is a method used in many studies to extract lipids from different organisms and it was chosen for this work because of the good results shown in the literature. However, in the case of *Nannochloropsis* sp., some modifications may be required to ensure an efficient and consistent lipid recovery. Various studies have reported that the presence of contaminants enhanced the accumulation of lipids in different microalgae species. For instance, Tang et al. studied the removal of PAR by *Coccomyxa subellipsoidea* coupled with the increase in lipid content, through acclimatization processes [71]. Another study reported the bioremediation of tannery effluent containing inorganic pollutants using *Nannochloropsis oculata* and verified a substantial lipid accumulation [78]. Shi et al. noted that in some microalgae species, enhancing the activity of antioxidant enzymes may be beneficial for lipid production [79]. In this context, it is known that exposure to CECs causes oxidative stress, and therefore, it is suggested that it leads to stimulation of lipid biosynthesis.

#### 3.3.2. FAME Profile

Species of the genus *Nannochloropsis* have different fatty acid distributions with C16, C18 and C20 being the most predominant. Their profile can be affected by several factors, including light exposure time, light intensity, temperature, pH and other environmental stresses, namely the presence of contaminants [28].

The method selected to identify the lipids present in the microalgae biomass after bioremediation included a series of mechanical cell disruption, solvent-based liquid extraction using chloroform and methanol and transesterification of fatty acids to fatty acid methyl esters (FAMEs). This conversion generated analytes more volatile and nonpolar which helps in the separation by GC-MS using a nonpolar column. It is noteworthy that the extraction and transesterification process can be carried out in a single step. However, this approach is limited in terms of the range of solvents that can be used for extraction, and it also results in a reduction in extraction efficiency. The process involving a multi-step process facilitates additional separation of lipid classes [59].

From the FAMEs collected from the control biomass (samples A1, A2 and A3) and from the biomass after bioremediation (samples B1, B2 and B3), GC-MS chromatograms were obtained. The FAMEs are separated by degree of saturation and size, with shorter-chain length fatty acids and more saturated fatty acids showing shorter retention times. Table 3 and Table 4 show the peaks obtained, corresponding to long chain FAMEs, with a retention times ranging from 18 to 23 min. In the mass chromatogram, characteristic peaks resulting from carbonic and ester group fragmentation were observed.

As demonstrated in Table 3, the main fatty acids present in the control are two hexadecadienoic acid isomers (both differ in the position and number of double bonds), palmitic acid, linoleic acid, oleic acid (in samples A1 and A2) and γ-linolenic acid (sample A3). Stearic acid was identified as main compound in samples A1 and A2. The mass chromatograms of the main compounds in sample A can be found in Supplementary data (Figures S1–S4). The presence of other peaks was also noticed at levels significantly lower than those described above, including eicosanoic acid (C20) in sample A3.

Following a 10-day exposure to the CECs, the fatty acid profile of *Nannochloropsis* sp. remained relatively stable. Compounds including hexadecadienoic acid isomers, palmitic acid, linoleic acid and γ-linolenic acid were identified in all samples B (Table 4). Notably, stearic acid and oleic acid were not identified in the chromatogram, suggesting that the exposure to these pollutants led to a decrease in their production. The mass chromatograms of the majority analytes can be found in Supplementary Data (Figures S5–S8).

The presence of peaks with retention times above 30 min corresponds to the peaks of siloxane present in the column. Furthermore, within minority peaks was probably identified IMID in sample B1 with a retention time of 17.83 min and IBU in sample B2 with a retention time of 14.71 min. As previously mentioned, despite being residual, these results suggest that these pollutants may have the ability to accumulate in the lipid part of the microalgae.

In future work, a quantification of these fatty acids will be able to establish a correlation between the exposure to CECs and the biosynthesis of specific fatty acids that are relevant to a wide range of multiple applications beyond biodiesel production.

## 4. Conclusions

The study presented in this article aimed to explore *Nannochloropsis* sp. in a circular approach to bioremediate PAR, BPA, IMID, MP, TCS and IBU from a synthetic wastewater, while evaluating the valorisation of the produced biomass through lipid extraction and FAME analysis.

*Nannochloropsis* sp. showed resilience in surviving in the presence of the mixture of CECs at 10 µg mL^−1^ under experimental conditions, elucidating their potential to grow under environmental relevant concentrations and promise to be efficient for water bioremediation processes in real-life scenarios.

Significant removals were obtained from the microalgae system. The highest efficiency was obtained for MP (100%) from an initial concentration of 10.43 µg mL^−1^, followed by BPA, with a total removal of 93 ± 2% from an initial concentration of 10.43 µg mL^−1^. IMID reached a removal efficiency of 20 ± 2% from an initial concentration of 10.30 µg mL^−1^. PAR and IBU achieved total removals of 64 ± 2% and 49 ± 5% from initial concentrations of 11.16 µg mL^−1^ and 8.03 µg mL^−1^, respectively. In addition to abiotic factors, *Nannochloropsis* sp. was shown to play a key role in the removal of the CECs, highlighting its potential for effective bioremediation in real-life scenarios.

The GC-MS analysis demonstrated the presence of mainly FAMEs, both in sample A (control) and B (microalgae with pollutants). The analytical technique identified in the control samples via the presence of hexadecadienoic acid isomers (C16:2), palmitic acid (C16), linoleic acid (C18:2), oleic acid (C18:1), stearic acid (C18) and γ-linolenic acid (C18:3) in different proportions. After the exposure to CECs, oleic acid and stearic acid were not identified in the chromatograms, suggesting that the pollutants affected their production in microalgae. To close the circle of this process, lipids can be valorised for various applications. The potential presence of CECs or hazardous degradation products in the lipids should be carefully evaluated and considered to ensure that the final product is in compliance with applicable regulatory policies and quality standards. The generation of biodiesel as a clean energy source from *Nannochloropsis* fatty acids is widely studied; on the other hand, oil-derived plasticizers and surfactants can also be obtained from microalgae lipids and used as building blocks for bio-based thermosetting materials or foams [80,81,82].

To sum, these results support the great potential of *Nannochloropsis* sp. for sustainable bioremediation and lipid valorisation for various applications, actively contributing to the circular economy.

## Figures and Tables

**Table 1:** Chemical structure of each contaminant, and relevant physico-chemical characteristics. (“-“: no information available).

Contaminant	Chemical Structure	CEC Category	ED List	pK_a_	Solubility (mg L^−1^)	Log K_ow_	Henry’s Law Constant
Paracetamol	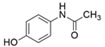	Pharmaceutical	-	9.4	14,000	0.46	8.8 × 10^−10^
Ibuprofen	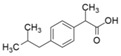	Pharmaceutical	-	4.9	21	3.97	1.7 × 10^−7^
Bisphenol A	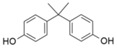	Industrial chemical	I	9.6	300	3.32	4 × 10^−11^
Methylparaben	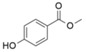	Preservative	II	8.5	28	1.96	2.3 × 10^−9^
Triclosan	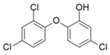	Antimicrobial	II	7.9	10	4.76	2.1 × 10^−8^
Imidacloprid	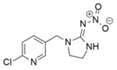	Pesticide	-	1.6/11.1	580	0.57	6.5 × 10^−11^

**Table 2:** Multiple gradient chromatographic analysis of eluents A and B.

Time (min)	Eluent A (%)	Eluent B (%)	Flow Rate (mL min^−1^)
0	50	50	0.6
5	80	20	0.6
15	100	0	0.6
17	100	0	0.6

**Table 3:** Peaks identified by gas chromatography and the corresponding fatty acids (A: microalgae in f2 medium without CECs).

Sample	Retention Time	FAMEs	Molecular Formula	Molecular Weight (amu)	Area (%)
A1	18.954	Hexadecadienoic acid methyl ester (isomer)	C_17_H_30_O_2_	266.2	31.01
	19.023	Hexadecadienoic acid methyl ester (isomer)	C_17_H_28_O_2_	264.2	48.46
	19.269	Palmitic acid methyl ester	C_17_H_34_O_2_	270.2	72.38
	20.922	Linoleic acid methyl ester	C_19_H_34_O_2_	294.2	61.13
	20.979	Oleic acid methyl ester	C_19_H_36_O_2_	296.3	100.00
	21.174	Stearic acid methyl ester	C_19_H_38_O_2_	298.3	10.15
A2	18.948	Hexadecadienoic acid methyl ester (isomer)	C_17_H_30_O_2_	266.2	25.90
	19.017	Hexadecadienoic acid methyl ester (isomer)	C_17_H_32_O_2_	268.2	41.69
	19.269	Palmitic acid methy ester	C_17_H_34_O_2_	270.2	67.19
	20.917	Linoleic acid methyl ester	C_19_H_34_O_2_	294.3	55.93
	20.985	Oleic acid methyl ester	C_19_H_36_O_2_	296.3	100.00
	21.168	Stearic acid methyl ester	C_19_H_38_O_2_	298.3	11.63
A3	18.960	Hexadecadienoic acid methyl ester (isomer)	C_17_H_30_O_2_	266.2	22.27
	19.040	Hexadecadienoic acid methyl ester (isomer)	C_17_H_28_O_2_	264.2	67.31
	19.280	Palmitic acid methy ester	C_17_H_34_O_2_	270.2	64.77
	20.922	Linoleic acid methyl ester	C_19_H_34_O_2_	294.3	43.39
	21.020	γ-linolenic acid methyl ester	C_19_H_32_O_2_	292.2	100.00
	23.039	Eicosanoic acid methyl ester	C_21_H_42_O_2_	306.3	12.53

**Table 4:** Peaks identified by gas chromatography and the corresponding fatty acids (B: microalgae in f2 medium with CECs).

Sample	Retention Time	FAMEs	Molecular Formula	Molecular Weight (amu)	Area (%)
B1	18.954	Hexadecadienoic acid methyl ester (isomer)	C_17_H_30_O_2_	266.2	35.08
	19.023	Hexadecadienoic acid methyl ester (isomer)	C_17_H_28_O_2_	264.2	82.40
	19.257	Palmitic acid methy ester	C_17_H_34_O_2_	270.2	65.30
	20.911	Linoleic acid methyl ester	C_19_H_34_O_2_	294.2	39.25
	20.985	γ-linolenic acid methyl ester	C_19_H_32_O_2_	292.2	100.00
B2	18.954	Hexadecadienoic acid methyl ester (isomer)	C_17_H_30_O_2_	266.2	44.76
	19.028	Hexadecatrienoic acid methyl ester (isomer)	C_18_H_28_O_2_	278.3	69.00
	19.257	Palmitic acid methy ester	C_17_H_34_O_2_	270.2	60.31
	20.916	Linoleic acid methyl ester	C_19_H_34_O_2_	294.3	62.44
	20.991	γ-linolenic acid methyl ester	C_19_H_32_O	292.2	100.00
B3	18.948	Hexadecadienoic acid methyl ester (isomer)	C_17_H_30_O_2_	266.2	32.32
	19.017	Hexadecadienoic acid methyl ester (isomer)	C_17_H_28_O_2_	264.2	67.10
	19.246	Palmitic acid methy ester	C_17_H_34_O_2_	270.2	60.72
	20.905	Linoleic acid methyl ester	C_19_H_34_O	294.3	36.71
	20.974	γ-linolenic acid methyl ester	C_19_H_32_O	292.2	100.00

## Data Availability

The raw data supporting the conclusions of this article will be made available by the authors on request.

## Supplementary Materials

The following supporting information can be downloaded at: https://www.mdpi.com/article/10.3390/bioengineering12030246/s1, Figure S1: Chromatogram peaks of experiment A1 (the transesterification of lipids was extracted from negative control A1); Figure S2: Chromatogram peaks of experiment A2 (the transesterification of lipids was extracted from negative control A2). Figure S3: Chromatogram peaks of experiment A3 (the transesterification of lipids was extracted from negative control A3). Figure S4: Mass chromatograms of the majority compounds identified in samples A1, A2 and A3. Figure S5: Chromatogram peaks of experiment B1 (the transesterification of lipids was extracted from bioremediation after 10 days exposed to CECs B1). Figure S6: Chromatogram peaks of experiment B2 (the transesterification of lipids was extracted from bioremediation after 10 days exposed to CECs B2). Figure S7: Chromatogram peaks of experiment B3 (the transesterification of lipids was extracted from bioremediation after 10 days exposed to CECs B3). Figure S8: Mass chromatograms of the majority compounds identified in samples B1, B2 and B3.
