# Supplementary material for: Bioremediation of Synthetic Wastewater with Contaminants of Emerging Concern by Nannochloropsis sp. and Lipid Production: A Circular Approach
Source: Bioengineering (Basel). 2025 Feb 28;12(3):246. doi: 10.3390/bioengineering12030246 (PMC11939778; doi:10.3390/bioengineering12030246)

Supplementary data

Figure S1. Chromatogram Peaks of experiment A1 (the transesterification of lipids was extracted from negative control A1).

Chromatogram Peaks

Peak	Start	RT	End	Height	Area	Area %
1	3.307	3.356	3.407	129202	237438	1.42
2	14.563	14.605	14.685	113860	209849	1.26
3	14.760	14.800	14.880	162568	239912	1.44
4	17.106	17.134	17.197	148558	220257	1.32
5	18.153	18.204	18.273	90506	215107	1.29
6	18.427	18.456	18.494	238834	344960	2.07
7	18.651	18.679	18.725	457655	667195	4.00
8	18.771	18.817	18.851	121437	315888	1.89
9	18.908	18.954	18.988	3180832	5174420	31.01
10	18.988	19.023	19.120	4148522	8086113	48.46
11	19.200	19.269	19.332	6694512	12077628	72.38
12	20.308	20.339	20.373	123336	192564	1.15
13	20.544	20.573	20.596	175404	249394	1.49
14	20.860	20.922	20.945	5454700	10200847	61.13
15	20.945	20.979	21.114	6364105	16685943	100.00
16	21.140	21.174	21.214	1173586	1693193	10.15
17	21.214	21.248	21.282	84528	168687	1.01
18	21.634	21.672	21.723	128721	202877	1.22
19	22.719	22.765	22.828	62521	170523	1.02
20	22.991	23.034	23.125	458007	858069	5.14
21	37.820	38.111	38.306	17285	263401	1.58
22	40.423	41.413	42.019	45477	1917654	11.49

Figure S2. Chromatogram Peaks of experiment A2 (the transesterification of lipids was extracted from negative control A2).

Chromatogram Peaks

Peak	Start	RT	End	Height	Area	Area %
1	3.311	3.350	3.412	170155	291494	1.21
2	17.090	17.129	17.201	198884	282322	1.17
3	18.410	18.450	18.488	206830	297778	1.23
4	18.554	18.616	18.645	155876	313313	1.30
5	18.645	18.674	18.742	402662	587777	2.43
6	18.755	18.811	18.845	167264	410345	1.70
7	18.903	18.948	18.983	3835805	6255267	25.90
8	18.983	19.017	19.114	5154184	10070051	41.69
9	19.189	19.269	19.320	7892316	16229657	67.19
10	20.300	20.327	20.384	199186	372552	1.54
11	20.534	20.568	20.590	238852	372009	1.54
12	20.590	20.613	20.699	168924	301570	1.25
13	20.848	20.917	20.940	6367932	13509025	55.93
14	20.940	20.985	21.105	8939212	24154746	100.00
15	21.123	21.168	21.208	1856216	2808115	11.63
16	21.208	21.249	21.346	124429	275904	1.14
17	21.627	21.660	21.706	291860	430002	1.78
18	22.980	23.028	23.097	788940	1465066	6.07
19	39.307	39.582	39.639	39301	743866	3.08
20	39.934	40.028	40.446	55203	854741	3.54
21	40.446	40.772	41.241	100947	2122462	8.79

Figure S3. Chromatogram Peaks of experiment A3 (the transesterification of lipids was extracted from negative control A3).

Chromatogram Peaks

Peak	Start	RT	End	Height	Area	Area %
1	3.316	3.344	3.423	170934	347571	1.11
2	14.566	14.599	14.662	198232	349679	1.11
3	14.771	14.800	14.834	436243	610199	1.94
4	17.106	17.134	17.169	400819	556952	1.77
5	18.147	18.204	18.267	222944	497965	1.58
6	18.427	18.456	18.498	442957	663303	2.11
7	18.651	18.679	18.771	789361	1301812	4.14
8	18.777	18.817	18.857	271300	436920	1.39
9	18.914	18.960	18.988	3662339	7003102	22.27
10	18.988	19.040	19.091	8261586	21164132	67.31
11	19.091	19.097	19.126	490526	587553	1.87
12	19.200	19.280	19.332	9416213	20363615	64.77
13	19.715	19.738	19.779	234032	363884	1.16
14	20.070	20.115	20.149	268576	434184	1.38
15	20.305	20.333	20.412	359437	658565	2.09
16	20.867	20.922	20.957	5823978	13642943	43.39
17	20.957	21.020	21.082	10863690	31441382	100.00
18	21.134	21.174	21.208	1485789	2306731	7.34
19	21.208	21.254	21.294	323789	757670	2.41
20	21.391	21.437	21.466	122335	326843	1.04
21	21.629	21.666	21.706	505799	798960	2.54
22	21.935	21.975	22.032	270255	479177	1.52
23	22.982	23.039	23.119	2018704	3940019	12.53
24	40.068	40.160	40.297	122942	622074	1.98
25	41.007	41.132	41.224	49737	333105	1.06
26	42.416	42.517	42.614	69236	391213	1.24

Figure S4. Mass chromatograms of the majority compounds identified in samples A1, A2 and A3.

Hexadecadionoic acid methyl ester (isomer)

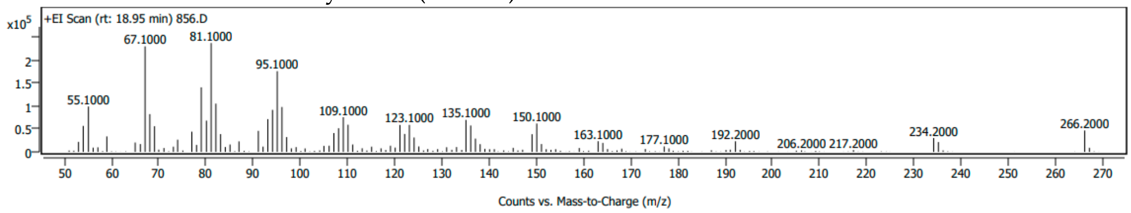

Hexadecadionoic acid methyl ester (isomer)

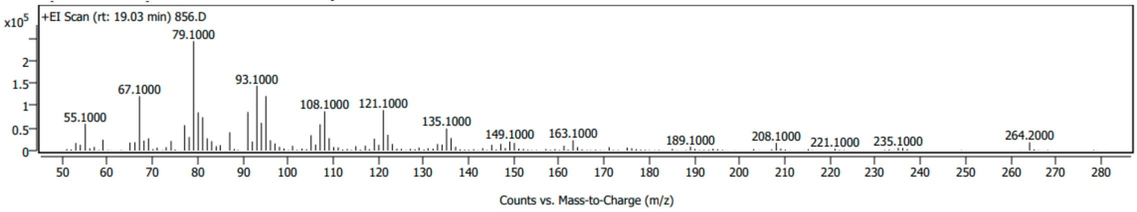

Palmitic acid methyl ester

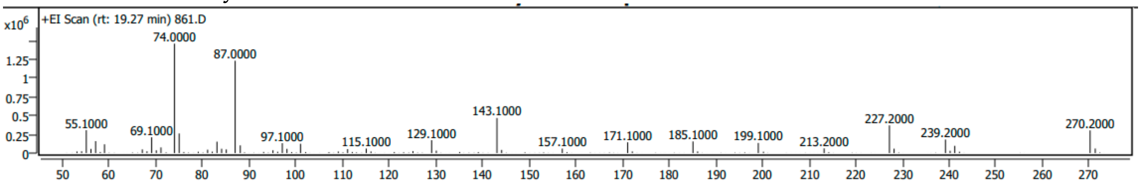

Linoleic acid methyl ester

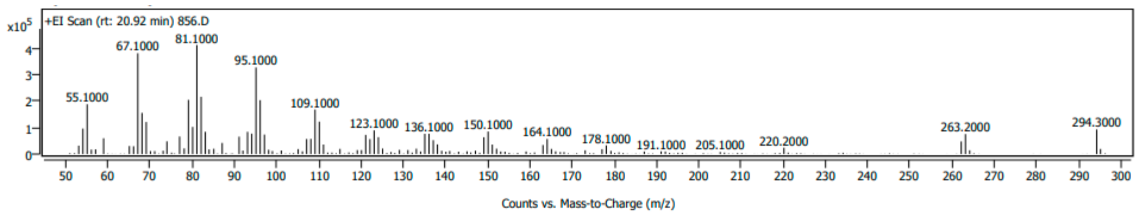

Oleic acid methyl ester

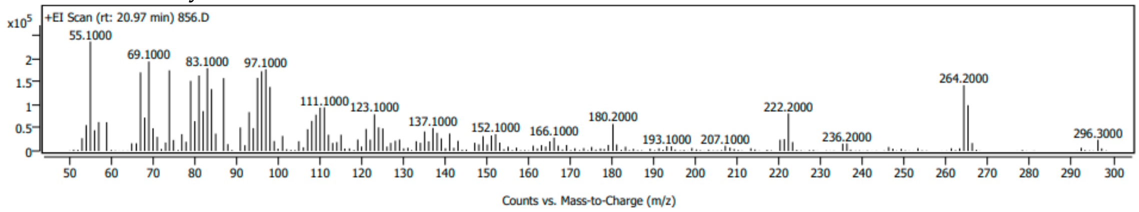

γ-Linolenic acid methyl ester

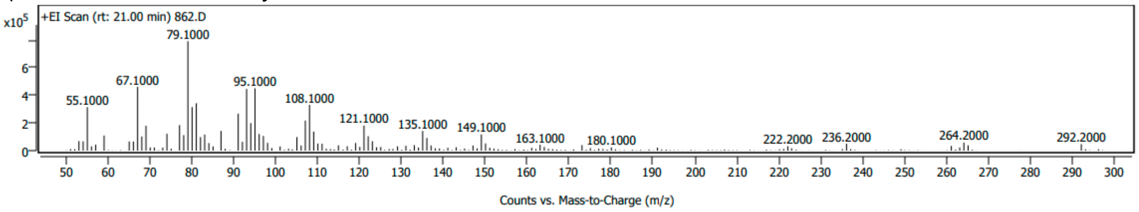

Stearic acid methyl ester

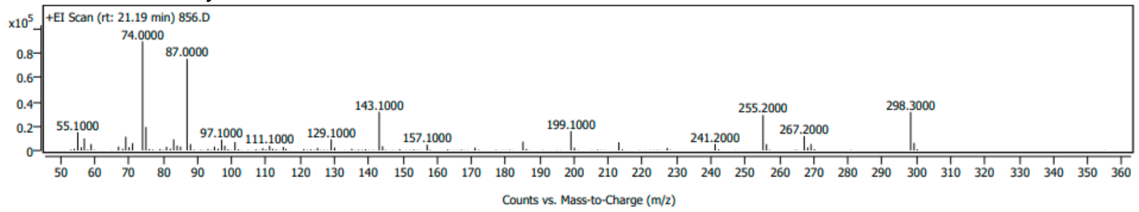

Eicosanoic acid methyl ester

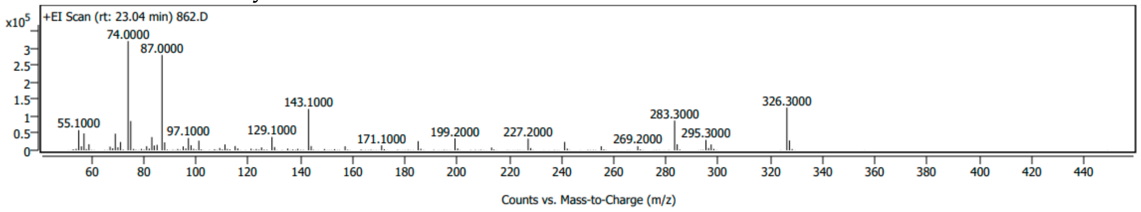

Figure S5. Chromatogram Peaks of experiment B1 (the transesterification of lipids was extracted from bioremediation after 10 days exposed to CECs B1).

Chromatogram Peaks

Peak	Start	RT	End	Height	Area	Area %
1	3.317	3.356	3.411	153563	222439	2.84
2	16.591	16.631	16.665	43538	81075	1.04
3	18.137	18.210	18.256	85923	195845	2.50
4	18.433	18.462	18.496	202744	293335	3.75
5	18.596	18.628	18.651	88080	117690	1.50
6	18.651	18.685	18.758	394879	586948	7.49
7	18.772	18.822	18.851	122044	184723	2.36
8	18.908	18.954	18.988	1763981	2747386	35.08
9	18.988	19.023	19.086	3415049	6453539	82.40
10	19.086	19.097	19.126	88972	123554	1.58
11	19.171	19.211	19.223	163569	239003	3.05
12	19.223	19.257	19.326	3356881	5114179	65.30
13	19.458	19.503	19.537	98431	153113	1.95
14	19.715	19.744	19.784	106407	162121	2.07
15	20.078	20.121	20.151	88257	134727	1.72
16	20.305	20.339	20.424	168801	293499	3.75
17	20.861	20.911	20.939	1971700	3074482	39.25
18	20.939	20.985	21.060	4027424	7832357	100.00
19	21.140	21.174	21.203	207122	303885	3.88
20	21.392	21.443	21.512	78230	217086	2.77
21	21.639	21.672	21.709	207825	304355	3.89
22	22.994	23.039	23.125	320707	568504	7.26
23	36.183	36.847	37.379	28428	1103646	14.09
24	38.432	39.862	40.715	33477	2568709	32.80

Figure S6. Chromatogram Peaks of experiment B2 (the transesterification of lipids was extracted from bioremediation after 10 days exposed to CECs B2).

Chromatogram Peaks

Peak	Start	RT	End	Height	Area	Area %
1	3.312	3.344	3.402	152226	249630	2.21
2	18.153	18.210	18.263	113763	258282	2.29
3	18.427	18.462	18.495	253557	377214	3.34
4	18.594	18.628	18.651	116365	162432	1.44
5	18.651	18.685	18.759	506341	769971	6.82
6	18.771	18.822	18.857	165884	254375	2.25
7	18.902	18.954	18.988	3159802	5055546	44.76
8	18.988	19.028	19.085	4036264	7794417	69.00
9	19.171	19.211	19.223	198857	306960	2.72
10	19.223	19.257	19.320	4200614	6812925	60.31
11	19.457	19.503	19.532	144262	236502	2.09
12	19.532	19.566	19.595	57202	142754	1.26
13	19.715	19.743	19.824	154736	274995	2.43
14	20.070	20.115	20.155	130951	211160	1.87
15	20.201	20.236	20.298	53660	130473	1.16
16	20.298	20.339	20.436	259331	461176	4.08
17	20.865	20.916	20.945	4010977	7052926	62.44
18	20.945	20.991	21.082	5549042	11295586	100.00
19	21.128	21.174	21.208	342232	512839	4.54
20	21.208	21.248	21.284	62782	143912	1.27
21	21.391	21.443	21.498	59970	160396	1.42
22	21.639	21.672	21.705	332480	492133	4.36
23	22.996	23.039	23.124	479829	850826	7.53
24	38.374	39.255	39.821	19927	991653	8.78
25	40.123	41.430	42.402	71367	4379300	38.77

Figure S7. Chromatogram Peaks of experiment B3 (the transesterification of lipids was extracted from bioremediation after 10 days exposed to CECs B3).

Chromatogram Peaks

Peak	Start	RT	End	Height	Area	Area %
1	3.317	3.350	3.402	140100	218651	4.14
2	16.522	16.556	16.585	51079	74886	1.42
3	16.585	16.625	16.662	28680	58791	1.11
4	18.147	18.204	18.244	48323	107200	2.03
5	18.428	18.456	18.490	93437	136462	2.58
6	18.596	18.622	18.651	55314	79710	1.51
7	18.651	18.679	18.742	183210	278336	5.27
8	18.774	18.817	18.843	63725	93334	1.77
9	18.902	18.948	18.977	1129747	1707038	32.32
10	18.977	19.017	19.040	2256268	3543216	67.10
11	19.040	19.051	19.120	467238	714555	13.53
12	19.171	19.200	19.217	106488	152453	2.89
13	19.217	19.246	19.355	2137854	3206652	60.72
14	19.446	19.498	19.532	63397	96267	1.82
15	19.709	19.738	19.778	65162	102182	1.93
16	20.075	20.116	20.147	46728	69333	1.31
17	20.200	20.236	20.287	21044	53340	1.01
18	20.295	20.333	20.413	86685	164508	3.12
19	20.860	20.905	20.934	1215083	1938667	36.71
20	20.934	20.974	21.071	2965880	5280881	100.00
21	21.128	21.168	21.197	142757	213655	4.05
22	21.197	21.243	21.279	29690	74180	1.40
23	21.392	21.437	21.500	58973	146323	2.77
24	21.633	21.666	21.698	121246	177025	3.35
25	22.994	23.028	23.097	183527	348622	6.60
26	31.468	33.076	33.848	26059	2152421	40.76
27	38.386	39.393	39.822	25607	1281671	24.27

Figure S8. Mass chromatograms of the majority compounds identified in samples B1, B2 and B3.

Hexadecadionoic acid methyl ester (isomer)

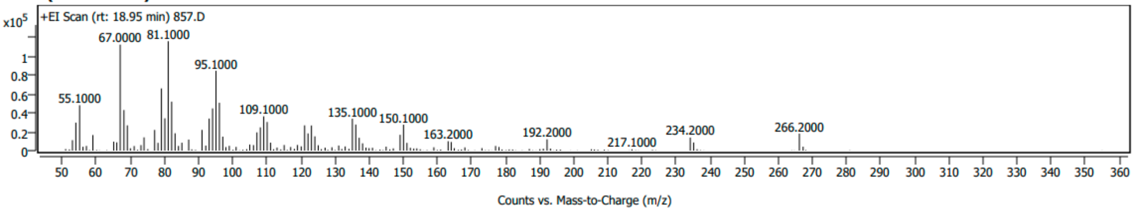

Hexadecadionoic acid methyl ester (isomer)

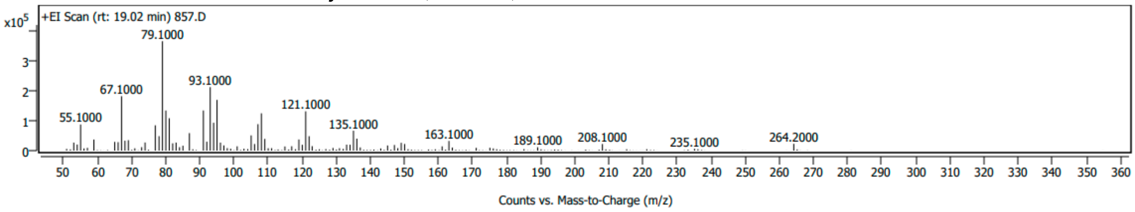

Hexadecatrienoic acid methyl ester (isomer)

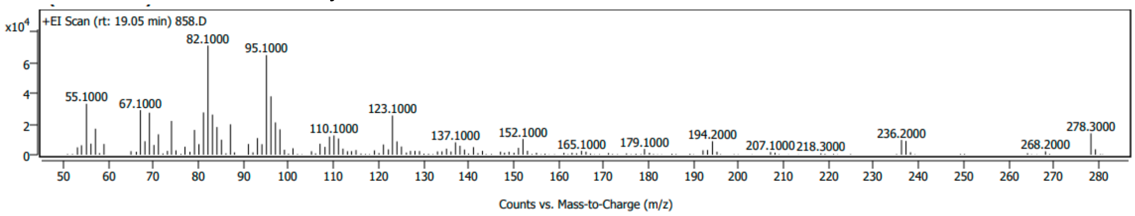

Palmitic acid methyl ester

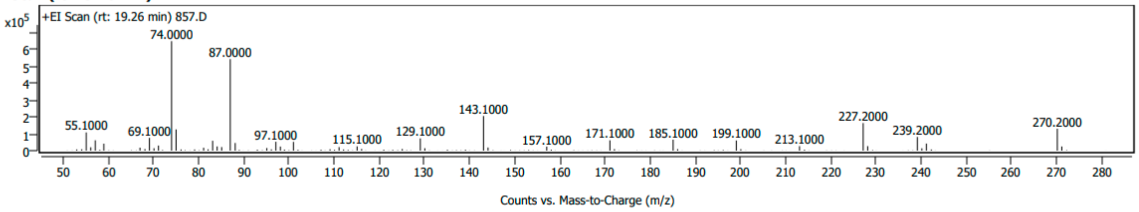

Linoleic acid methyl ester

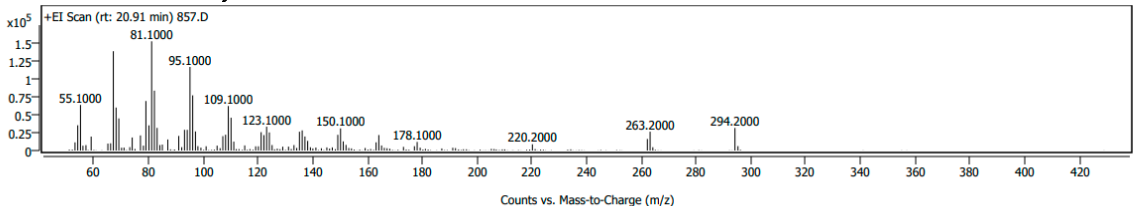

γ-Linolenic acid methyl ester

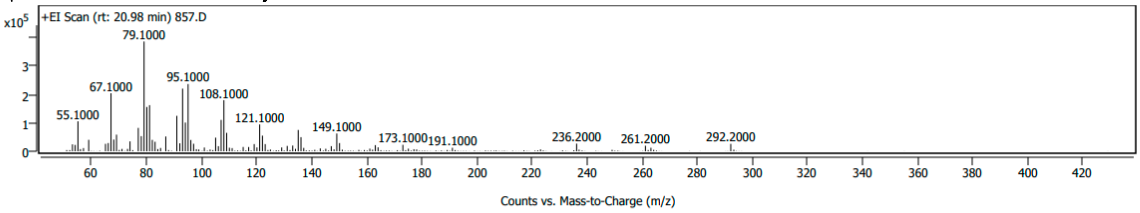

Supplement: Supplementary file 1 [file bioengineering-12-00246-s001.zip › bioengineering-3463728-supplementary.pdf]
